# Progress and Prospects in Metallic Fe*_x_*GeTe_2_ (3 ≤ *x* ≤ 7) Ferromagnets

**DOI:** 10.3390/molecules28217244

**Published:** 2023-10-24

**Authors:** Hongtao Ren, Mu Lan

**Affiliations:** 1 School of Materials Science and Engineering, Liaocheng University, Liaocheng 252000, China; 2 College of Optoelectronic Engineering, Chengdu University of Information Technology, Chengdu 610225, China

**Keywords:** Mermin-Wanger theory, Fe stoichiometry, strain, light control, electrical control, doping engineering, twisting, patterning, magnetic skyrmions, spin-orbit torque

## Abstract

Thermal fluctuations in two-dimensional (2D) isotropy systems at non-zero finite temperatures can destroy the long-range (LR) magnetic order due to the mechanisms addressed in the Mermin-Wanger theory. However, the magnetic anisotropy related to spin–orbit coupling (SOC) may stabilize magnetic order in 2D systems. Very recently, 2D Fe*_x_*GeTe_2_ (3 ≤ *x* ≤ 7) with a high Curie temperature (*T_C_*) has not only undergone significant developments in terms of synthetic methods and the control of ferromagnetism (FM), but is also being actively explored for applications in various devices. In this review, we introduce six experimental methods, ten ferromagnetic modulation strategies, and four spintronic devices for 2D Fe*_x_*GeTe_2_ materials. In summary, we outline the challenges and potential research directions in this field.

## 1. Introduction

The Mermin-Wanger theory [1,2] asserts that thermal fluctuations occur in 2D isotropy systems at non-zero finite temperatures, which destroy the long-range magnetic order (LRMO). Specifically, exchange interactions alone should not generate magnetic order in 2D systems, and magnetic anisotropy [3,4,5] is also needed to maintain the LRMO. Surprisingly, it was found experimentally that low-temperature long-range ferromagnetic order (LRFO) can exist in the Cr_2_Ge_2_Te_6_ monolayer [4] and CrI_3_ monolayer [5,6]. Soon after, a vast range of 2D magnetic systems, including metallic (Fe_3_GeTe_2_ (FGT) [7,8,9,10,11,12,13,14,15,16,17,18,19,20,21,22,23]), semiconductors (Cr_2_Ge_2_Te_6_ [4,24,25,26,27,28,29,30,31,32], CrI_3_ [5,33]), and topological insulators (MnBi_2_Te_4_ [34]), were successively implemented to promote the development of spintronics.

**Figure 1:**
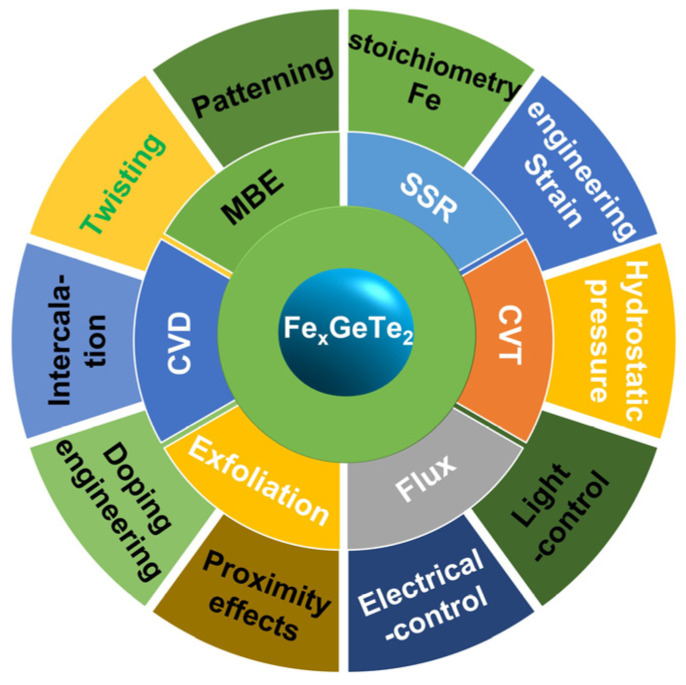
Overview of six synthesis methods and ten strategies for ferromagnetic Fe*_x_*GeTe_2_ (3 ≤ *x* ≤ 7) materials. Black font represents the synthesis methods and tunable strategies for obtaining the RTFM; white font represents the synthesis methods and strategies that can tune magnetism in experiments; green font represents only theoretically achievable tunable strategies.

Recently, Fe*_x_*GeTe_2_ (3 ≤ *x* ≤ 7) has received intense attention as a metallic and high Curie temperature (*T_C_*) ferromagnet. Six synthesis methods, including solid-state reaction (SSR) [35,36], chemical vapor transport (CVT) [8,13,37], the flux method [11,21,38,39,40,41,42,43,44,45], exfoliation [14,15,34,46,47,48,49,50], chemical vapor deposition (CVD) [51,52], and molecular beam epitaxy (MBE) [7,53,54,55,56,57,58], have been used to attempt to obtain wafer-scale Fe*_x_*GeTe_2_ (3 ≤ *x* ≤ 7) materials with room-temperature ferromagnetism (RTFM). However, the *T_C_* of the MBE-prepared Fe*_x_*GeTe_2_ (3 ≤ *x* ≤ 7) samples (see Figure 1) ranges from 390 to 530 K, with FGT (*T_C_* ≈ 400 K) [56], Fe_4_GeTe_2_ (F4GT; *T_C_* ≈ 530 K) [59], and Fe_5_GeTe_2_ (F5GT; *T_C_* ≈ 390 K) [60]. Furthermore, RTFM has also been tuned with ten strategies: Fe stoichiometry [9,39,51,59,61,62,63,64,65], strain engineering [46,48,66,67,68,69,70,71,72,73,74,75], hydrostatic pressure [76,77,78,79,80,81], light control [53,82], electrical control [83,84], proximity effects [56,57,85,86,87,88,89], doping engineering [14,20,38,43,44,62,90,91,92,93,94,95,96,97,98,99,100,101,102,103,104,105,106], intercalation [107,108] or irradiation [109], twisting [110,111], and patterning [16]. So far, twisting (see Figure 1) has only regulated the magnetic order theoretically and has not been achieved experimentally. Moreover, four typical devices have also been fabricated based on FGT, magnetic tunnel junctions (MTJ) [112,113,114,115], tunneling spin valves [18,99,116,117], nonlocal spin valves [118], and spin–orbit torque devices [20,119], in order to enrich their physical properties and develop their spintronic applications.

In this review, we introduce the developments in and structures of metallic Fe*_x_*GeTe_2_ ferromagnets. Subsequently, we summarize six experimental methods, as shown in Figure 1; the early samples prepared using SSR, CVT, and the flux method are mainly bulk single crystals. In addition, magnetic skyrmions in Fe*_x_*GeTe_2_ are summarized. Finally, we outline the challenges and potential research directions in this field.

## 2. Crystal Structure of Ferromagnetic Fe*_x_*GeTe_2_

**Figure 2:**
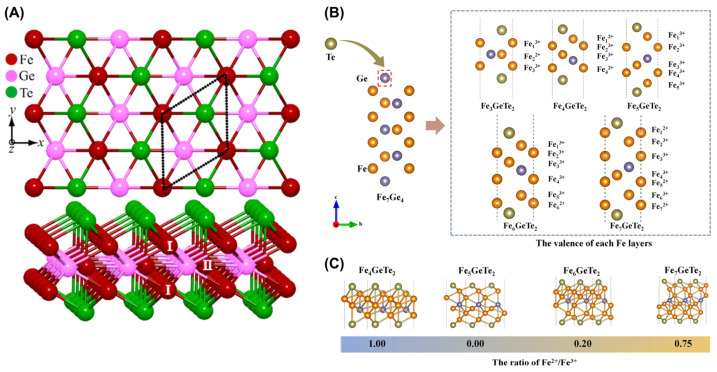
Crystal structure of Fe*_x_*GeTe_2_ (3 ≤ *x* ≤ 7). (**A**) Crystal structure of FGT monolayer. Inequivalent Fe sites are numbered by I and II. Reprinted with permission from [46]. Copyright 2016, American Physical Society. (**B**) Schematic illustration of Te–substituted Fe_7_Ge_4_ crystal and of five structures in the series Fe*_x_*GeTe_2_ (4 ≤ *x* ≤ 7). (**C**) Stacked plane views along the [001] direction of Fe*_x_*GeTe_2_. Reprinted with permission from [64]. Copyright 2022, Springer Nature.

The Fe_3_GeTe_2_ monolayer [46] comprises five atomic layers, as shown in Figure 2A. Specifically, Te atoms are located in the bottom and top layers, while Fe (I) atoms are located in the second and fourth layers. Notably, the intermediate layer is composed of Fe (II) atoms and Ge atoms. The local magnetic moments of Fe atoms for the FGT monolayer were determined by DFT-LDA (density functional theory–local density approximation) to be 1.723 *µ*_B_ and 1.005 *µ*_B_, with the out-of-plane direction being its easy axis. They may be related to the several partially occupied *d*-bands passing through the Fermi level. In addition, the number ratio of Fe^3+^ to Fe^2+^ in 2D Fe*_x_*GeTe_2_ [64] is related to the *x* value, as shown in Figure 2B. When the value of *x* is 3, the ratio of Fe^3+^/Fe^2+^ is 2:1. However, when the *x* value is 5, only Fe^3+^ is present (Figure 2C).

## 3. Synthesis of Metallic Fe*_x_*GeTe_2_ with FM

### 3.1. Solid-State Reaction (SSR)

**Figure 3:**
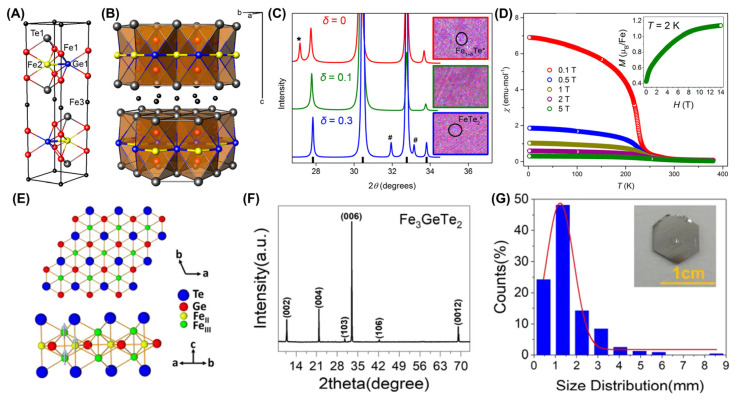
SSR–prepared FGT single crystals and polycrystalline structures. Fe_2.9_GeTe_2_ crystal structure: (**A**) unit cell and (**B**) polyhedral representation. The Fe3 position is vacant and shown only for comparison. (**C**) X–ray diffraction (XRD) patterns and energy–dispersive X–ray spectroscopy (EDXS) composition maps. * represents a second Fe_1+δ_Te phase; # represents an impurity FeTe_2_ phase. On the composition maps, Fe is presented in red, Ge in green, and Te in blue. (**D**) Magnetic susceptibility *χ* versus *T* plot in different applied fields. Reprinted with permission from [36]. Copyright 2015, American Chemical Society. (**E**) FGT crystal structure. (**F**) XRD. (**G**) Size distribution of the plate–like FGT single crystal. The inset is the optical photo of FGT. Reprinted with permission from [120]. Copyright 2022, American Chemical Society.

The solid-state reaction (SSR) is an experimental method for preparing bulk FGT crystals. As early as 2006, Deiseroth et al. [35] successfully prepared FGT crystals with hexagonal plates using SSR, which exhibited novel air stability and black metallic properties. Through magnetic testing, it was found that below 230 K, the crystals exhibited FM. Meanwhile, above 230 K, they exhibited Curie–Weiss paramagnetic behavior. After increased annealing, black Fe_3−δ_GeTe_2_ (0 < δ < 0.3) polycrystalline powders could be easily obtained with SSR. Fe1 and Ge1 atoms are shown in detail in different coordination environments in Figure 3A,B, and their unit cell consist of two layers. The lattice parameters increase monotonically with decreasing δ (it represents the degree of iron deficiency in FGT) (Figure 3C), but, when δ exceeds 0.3, FeTe_2_ will appear as an impurity phase. Its magnetic phase transition temperature (Figure 3D) is about 240 K. Furthermore, its saturation behavior (see inset in Figure 3D) slows down in high magnetic fields, which is different from ordinary ferromagnets.

In order to obtain large quantities of high-quality FGT single crystals, Li et al. [120] designed a new experimental method of solid-phase sintering followed by recrystallization (Figure 3E–G). The as-grown plate-like sample (~10 g) is a layered single crystal with a smooth and complete surface, and its size can reach up to 8.5 mm. By intercalating sodium into as-grown FGT, Weber et al. [107] raised its *T_C_* to 350 K. After intercalation, the sample retained obvious layered features, with edge lengths of a grain size ranging from 10–50 μm.

### 3.2. Chemical Vapor Transport (CVT)

**Figure 4:**
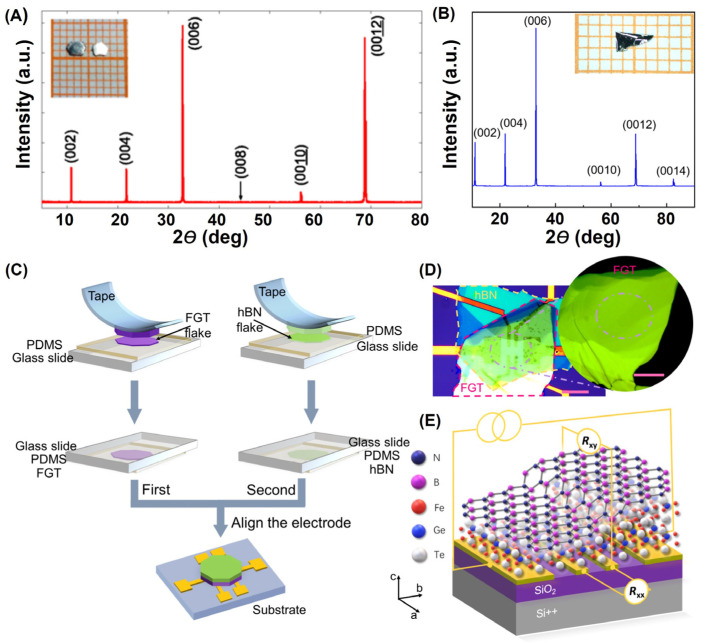
CVT-prepared FGT bulk single crystals. (**A**) XRD. The inset shows a photograph of FGT single crystals on a 1 mm grid. Reprinted with permission from [12]. Copyright 2017, American Physical Society. (**B**) XRD. The inset is the optical image of this FGT crystal. Reprinted with permission from [49]. Copyright 2021, American Physical Society. (**C**) Process of fabricating the nanodevices. (**D**) Thickness inhomogeneity in the FGT nanodevice (**E**). Reprinted with permission from [49]. Copyright 2021, American Physical Society.

One main difference from SSR is that CVT often uses iodine [8,13,14,15,17,37,39,61,119] or TeCl_4_ [12] as the transport agent, as shown in Figure 4. However, the samples obtained via SSR and CVT were both bulk single crystals, as shown in the inset of Figure 4A,B. Previous studies have mainly focused on the magnetic microstructures of quasi-2D FGT. Based on the prediction that FGT monolayer could be mechanically exfoliated [46], soon after, Chu et al. [15] and Zhang et al. [14], respectively, obtained FGT monolayer samples with the assistance of Au film and Al_2_O_3_, respectively. Actually, Zhang et al. [121] exfoliated FGT monolayer from the most possible cleaving planes (001), with a thickness of 1.75 nm and a nearest neighbor atomic spacing of 0.338 nm, which was highly consistent with the lattice constant (a = 0.399 nm; c = 1.63 nm) of the FGT crystal. However, thin layer FGT was highly prone to deteriorate in air, and the device fabrication processes (Figure 4C–E) needed to be carried out in a glove box [49]. Notably, many novel physics-related effects, such as patterning-induced RFTM [16], gate-tunable FM [14], and layer-dependent FM [15], have been discovered.

### 3.3. Flux Growth

The flux method [122,123,124] is commonly used to prepare single crystals. For example, Canfield et al. [122,123] grew a wide variety of single-crystal binary or ternary intermetallic compounds from molten flux solutions. However, the thickness and lateral size of the samples could not be accurately controlled, with mechanical exfoliation still required to obtain thinner samples when fabricating FGT devices.

**Figure 5:**
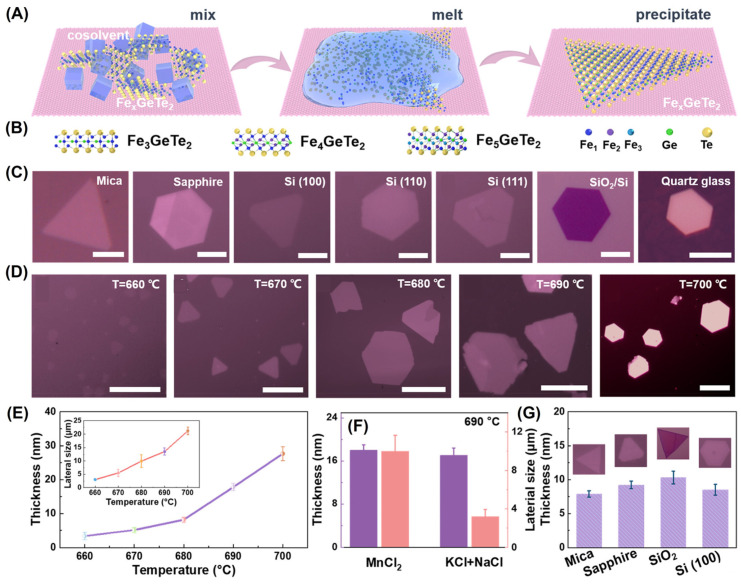
Fe*_x_*GeTe_2_ single crystal prepared via self–flux. (**A**) Schematic diagram of the flux-assisted growth process of Fe*_x_*GeTe_2_. (**B**) Cross–sectional structures of FGT, F4GT, and F5GT. (**C**) Optical images. Scale bars: 10 μm (Mica); 5 μm. (**D**) Optical images of FGT nanosheets grown on sapphire at 660, 670, 680, 690, and 700 °C, respectively. Scale bars: 10 μm. (**E**) Thickness and size evolution of FGT with temperature increase. (**F**) Differences in thicknesses and lateral sizes using different cosolvents. (**G**) Comparison of FGT under the same growth conditions. Reprinted with permission from [44]. Copyright 2022, American Chemical Society.

Recently, Gong et al. [44,45] proposed a universal flux-assisted growth (FAG) method (Figure 5A,B) to synthesize Fe*_x_*GeTe_2_ and M*_y_*Fe_5−*y*_GeTe_2_ (M = Co, Ni) nanosheets on various substrates (Figure 5C). In addition, the sample thickness and lateral size (Figure 5D) of FGT could be precisely controlled by the growth temperature (Figure 5E) or cosolvents (Figure 5F). Although the FGT samples with a thickness of 5–10 nm (Figure 5G) were prepared on various substrates, in order to obtain atomically thin materials (ATMs), a confinement environment must be provided through two substrates. Up to 80 layered and non-layered ATMs [45] have also been successfully synthesized using FAG, which provides a new strategy for preparing wafer-scale 2D materials.

### 3.4. Exfoliation

#### 3.4.1. Mechanical Exfoliation

Conventional mechanical exfoliation [125,126] can cleave thin FGT flakes onto SiO_2_/Si substrates, but its thinnest thickness is around 4.8 nm. After depositing Au onto SiO_2_/Si substrate, thinner samples can be obtained, and the Au substrate improves the yield to grow various thin layers of materials, including graphene [127,128], MoS_2_ [47,129,130,131,132], WSe_2_ [47,129,133], Bi_2_Te_3_ [129], and FGT [15,47]. Nevertheless, only a small amount of material can be exfoliated to a monolayer, which hinders the development of 2D magnetic materials. Notably, an Al_2_O_3_-assisted exfoliation method was also designed to produce monolayer FGT [14] and MnBi_2_Te_4_ [34] single crystals. When the sample was thinned from bulk to a monolayer, its *T_C_* decreased from 180 K to 20 K.

#### 3.4.2. Liquid-Phase Exfoliation

**Figure 6:**
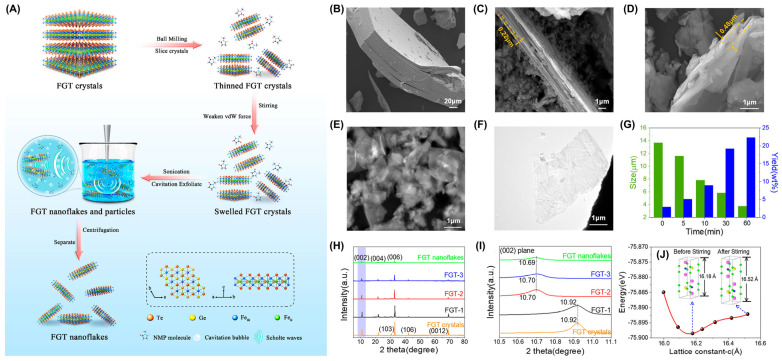
(**A**) Schematic diagram of TS–LPE. SEM images of FGT crystals (**B**) in a pristine state and after ball milling (**C**), stirring (**D**), and sonication (**E**). (**F**) TEM image of FGT nanoflakes after centrifugation. (**G**) Statistical graph of the influence of the ball–milling time on the center size and the yield. (**H**) XRD peaks and (**I**) enlarged view of (002) peaks. FGT−1, FGT−2, and FGT−3 are the samples after ball milling (**C**), stirring (**D**), and sonication (**E**), respectively. (**J**) Variation in the cell energy with the lattice constant in the c-axis direction. Reproduced with permission from [50]. Copyright 2022, American Chemical Society.

Although many methods including SSR [107], CVT [14,15,17,19,61,119,121,134], flux [39,43,44,45], and MBE [7,53,54,55,56,57,58,59,60] have been used to prepare 2D FGT, an economical method for the large-scale preparation of few- or single-layer FGT nanoflakes is still lacking. As a typical example, Ma et al. [50] developed three-stage sonication-assisted liquid-phase exfoliation (TS-LPE) (Figure 6A) to produce large semiconductive FGT nanoflakes. After ball milling, the sample size and thickness (Figure 6B,C) are reduced by the milling time (Figure 6G), exposing more boundaries. Stirring causes the interlayer spacing to expand (Figure 6C,D), weakening the interlayer force to facilitate detachment and obtain high-integrity nanoflakes. In addition, XRD analysis [135] (Figure 6H,I) reflects the evolution of interlayer spacing. The expansion of interlayer spacing causes the FGT unit cell to move away from the equilibrium state in the c-direction (Figure 6J), making them unstable and prone to spall. In practice, the oxidation on the surface layer altered the electronic structure of the FGT system, making the FGT sample semiconductive and different from the metallic FGT prepared using other methods.

### 3.5. Chemical Vapor Deposition (CVD)

So far, researchers have mainly used CVT to prepare 2D magnetic bulk single crystals, which are then exfoliated into atomic layers to prepare devices. However, poor control of the number of layers and a limited sample size have hindered the development of 2D magnets. As a typical example, Liu et al. [51] designed a confined space chemical vapor deposition (CS-CVD) method for preparing 2D FGT or F5GT ferromagnets. They found that the optimal growth temperature was 570–580 °C, with an optimal distance of 10 cm between the Fe/Ge precursor and the Te precursor. When the thickness of the F5GT flakes changed from 4 nm to 1 nm, the *T_C_* value decreased by 100K. Very recently, Liu et al. [52] also introduced a general competitive-chemical-reaction-controlled CVD method for producing FGT crystals. The sample was a single layer with a grain size of ~50 μm.

### 3.6. Molecular Beam Epitaxy (MBE)

**Figure 7:**
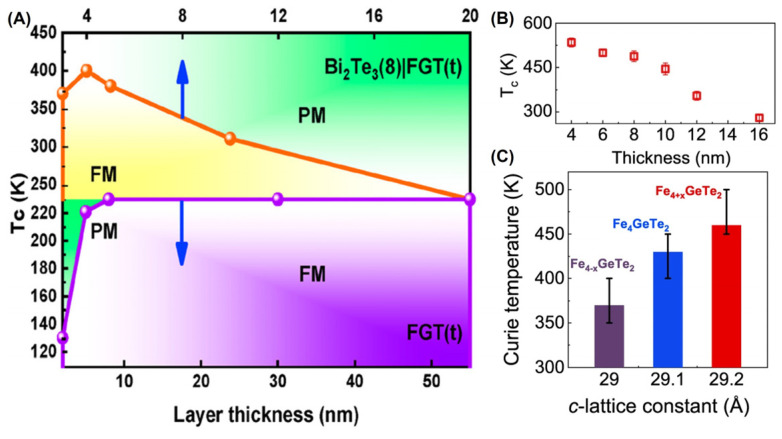
MBE–prepared FGT thin films. (**A**) Magnetic phase diagram of FGT and Bi_2_Te_3_|FGT *versus* layer thickness and temperature. Reproduced with permission from [56]. Copyright 2020, American Chemical Society. (**B**) *T_C_* for F4GT thin films with the thickness. (**C**) *T_C_ versus* the c–lattice constants of F4GT. Reproduced with permission from [59]. Copyright 2023, Springer Nature.

Wafer-scale single crystalline FGT thin films were grown on various substrates using the molecular beam epitaxial (MBE) [7,54,55,56,57,136] technique. After heterointegration with the topological insulator Bi_2_Te_3_ (Figure 7A), the T_C_ of FGT can be increased to 400 K. This enhancement may be related to the interface exchange coupling. Remarkably, when the thickness of F4GT decreases, its T_C_ is increased from 270 K to 530 K (Figure 7B). For F4GT thin films with a thickness of 10 nm, increasing the dosage of Fe can enhance their T_C_ (Figure 7C). Although MBE can alone prepare wafer-scale Fe*_x_*GeTe_2_ (3 ≤ *x* ≤ 7) materials with RTFM, this method requires a high vacuum environment, which makes it expensive and limits its industrial applications.

## 4. Controlling FM in Metallic Fe*_x_*GeTe_2_

### 4.1. Fe Stoichiometry

**Figure 8:**
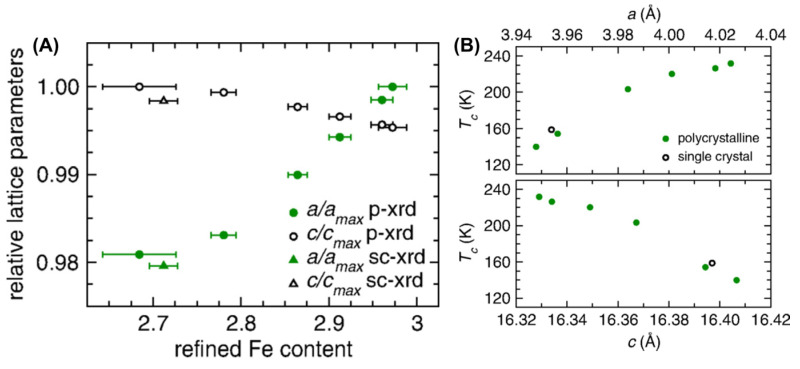
(**A**) Normalized lattice parameters as a function of refined Fe content for FGT samples (powder X-ray diffraction (p-xrd) at room temperature), including results obtained from single-crystal X-ray (sc-xrd) diffraction data collected at 173 K. (**B**) *T_C_* as a function of lattice parameters. Reprinted with permission from [9]. Copyright 2016, American Physical Society.

In this research area, the earliest discovery was that the FM in polycrystalline FGT bulk structures [9] was related to the Fe content (Figure 8). The higher the Fe content, the larger the lattice constant of the a-axis and the smaller the lattice constant of the *c*-axis (Figure 8A). Single crystal samples show similar results to the polycrystalline samples. Moreover, the T_C_ (Figure 8B) and M_S_ decreased with the decrease in Fe content. Subsequently, ferromagnetic F4GT [44,59,61] and F5GT [39,42,44,51,62,137,138,139,140,141,142,143,144] materials were also obtained in experiments.

However, most previous reports have focused on FGT materials with a single Fe stoichiometry, and there have been few studies on Fe*_x_*GeTe_2_ materials using the same experimental method. In addition, theoretical calculations [63] revealed that as the Fe content increased, the interlayer gap gradually increased, and the magnetic anisotropy of its monolayer changed from out-of-plane (FGT) to in-plane (F4GT and F5GT).

**Figure 9:**
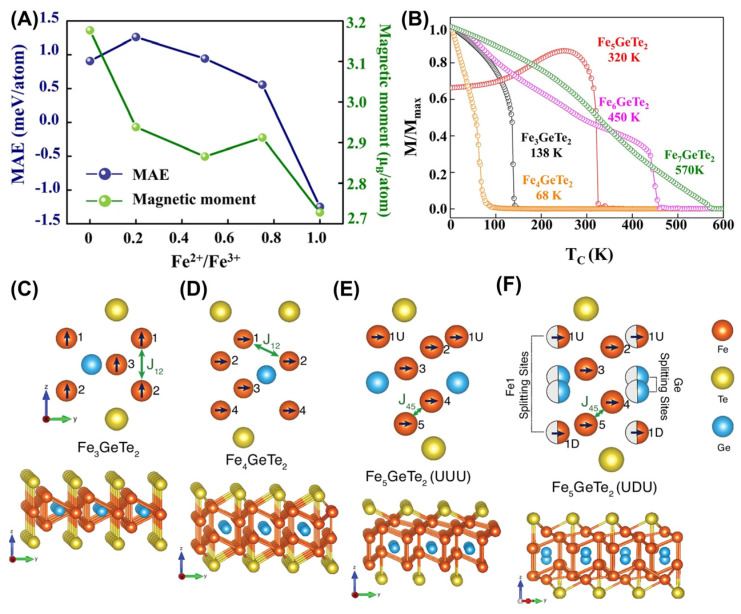
(**A**) Calculated magnetic anisotropy energy (MAE) and magnetic moment per atom for various Fe^2+^/Fe^3+^ ratios (*x*). (**B**) Calculated normalized magnetization of Fe atoms in Fe*_n_*GeTe_2_ as a function of temperature from Monte Carlo simulation. Reprinted with permission from [64]. Copyright 2022, Springer Nature. Crystal structure (**C**–**F**) of Fe*_x_*GeTe_2_ (3 ≤ *x* ≤ 5) monolayer. Side Views of the FGT (**C**), F4GT (**D**), UUU Fe5GT (**E**), and UDU F5GT monolayer (**F**). The half–colored circles in Figure 9F show the Fe1–Ge split sites present in the UDU configuration. The lower panel shows a 3D side view of the Fe*_x_*GeTe_2_ monolayers. Note that: $MAE=E_{⊥}-E_{∥}$. Reprinted with permission from [65]. Copyright 2023, Springer Nature.

Although previous reports have made significant progress in 2D Fe*_x_*GeTe_2_ systems, the mediation of magnetic anisotropy and their magnetic nature remains unresolved. Very recently, Liu et al. [64] preliminary reported a valence-dependent magnetic exchange model to explain the complex magnetic phase in Fe*_x_*GeTe_2_ systems. Furthermore, the magnetic moment and MAE (Figure 9A) were almost linearly correlated with Fe^2+^/Fe^3+^. Specifically, Fe^3+^ had a greater impact on magnetism, reducing the magnetic anisotropy energy in F5GT. Based on MAE and *J* (it represents the exchange coupling constant), the *T_C_* could be estimated using the 2D Heisenberg model. When x was greater than 4, the *T_C_* was much higher than RT (Figure 9B).

Moreover, the results obtained via different calculation methods [65] exhibit significant differences, especially when compared with experimental results. As *x* increases, its easy axis direction (shown by the black arrows) and the highest exchange interaction change from out-plane to in-plane (Figure 9C–F). Moreover, there were significant differences in the magnitude of the exchange interaction obtained via different calculation methods (GGA + DMFT, GGA, and GGA + U); specifically, the results calculated using GGA + U were overestimated. In addition, MAE exhibits a similar evolution from out-plane to in-plane. However, for different F5GT (UUU or UDU) configurations, the calculation results varied significantly. It was obvious that the *T_C_* calculated via GGA + DMFT was underestimated, while the result calculated by GGA was overestimated, compared to Figure 9B.

### 4.2. Strain Engineering

Strain engineering is an efficient strategy for modulating the FM of 2D materials [67,68,145]. However, previous theoretical works have focused on applying strain to FGT supercells by changing the lattice constants [46,70,71,73,146] and calculating the exchange coupling, magnetic anisotropy, and magnetic moment of strain through ab initio DFT. Furthermore, the T_C_ could be estimated according to mean field theory (MFT) [10,61,65,147,148,149], random phase approximation (PRA) [147,149], or Monte Carlo (MC) [148,149,150,151] simulation. Recently, Miao et al. [48] and Yan et al. [72] loaded FGT nanoflakes into a three-point-bending experimental setup and applied uniaxial tensile strain to the sample on a polyimide (PI) or polyvinyl alcohol (PVA)/polyethylene terephthalate (PET) flexible polymer substrate by moving the needle. Moreover, the magnitude of the applied strain could be calculated using the following formula [48,72,152]:

(1) $$\varepsilon =\frac{T}{2R}$$

where *T* and *R* are the film thickness and bending radius, respectively. Surprisingly, after 0.32% strain was applied, the coercivity increased by 150% [48], a far greater increase than the improvement in H_C_ generated by other traditional magnetic materials [153]. Most notably, its T_C_ could be increased to 400 K [72] via uniaxial strain, further promoting the development of wearable spintronic devices with low-energy consumption.

### 4.3. Hydrostatic Pressure

**Figure 10:**
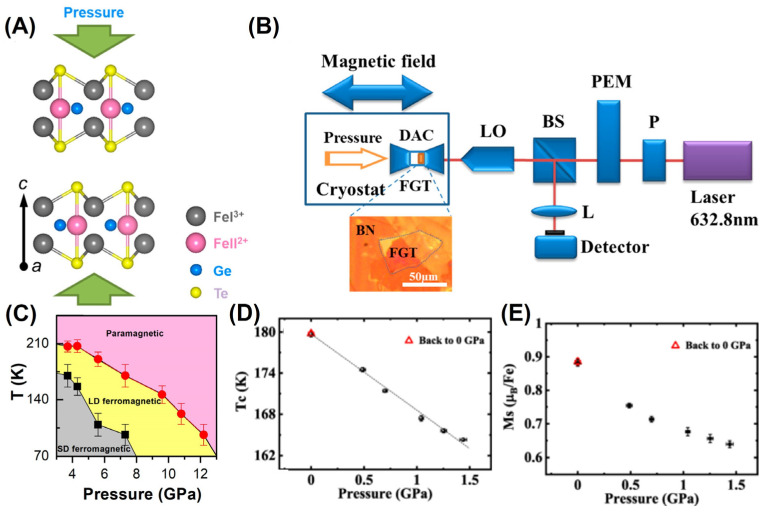
(**A**) Schematic of the lattice structure of FGT under pressure. (**B**) Schematic of in situ MCD experimental setup under hydrostatic pressure. The thin FGT sample was covered with a thick *h* BN to avoid degradation induced by pressure media. P: polarizer; PEM: photoelastic modulator; BS: beam splitter; DAC: diamond anvil cell; LO: 50× long-working distance objective; L: lens; FGT: thin FGT sample. (**C**) Pressure–dependent phase diagram. The gray region represents a single–domain (SD) ferromagnetic state, the yellow region represents a labyrinthine–domain (LD) ferromagnet, and the pink region shows the paramagnetic state. Reprinted with permission from [78]. Copyright 2019, American Chemical Society. (**D**) The pressure dependence of *T_C_*, where *T_C_* is estimated from the minimum point of *dM*/*dT*. (**E**) The variation in *Ms* for FGT is caused by increasing the pressure. Reprinted with permission from [79]. Copyright 2021, American Physical Society.

Tuning the exchange coupling and magneto-crystalline anisotropy by applying hydrostatic pressure is another commonly used method for regulating 2D magnetism, which has been achieved in Cr_2_Gr_2_Te_6_ [154,155], CrI_3_ [33,156], and FGT [77,78,79] systems (Figure 10). The ferromagnetic evolution of FGT nanosheets under different pressures can be revealed through in situ magnetic circular dichroism (MCD) spectroscopy (Figure 10B). Furthermore, the magnetic hysteresis loop at 30 K exhibited a rectangular shape below 7 GPa, while its loop presented an eight-shaped skewed shape above 7.3 GPa. Moreover, *T_C_* increases as the pressure further decreases (Figure 10C), which may be related to the strengthening of the exchange interactions.

As another typical example, *T_C_* and the magnetic moment also increased with the decrease in the hydrostatic pressure (Figure 10D,E). It is evident that the increasing pressure reduced the length of the Fe–Fe bond, which inhibited magnetization through modification of the exchange interactions. In addition, a monotonic relationship between *T_C_*, the magnetic moment, and pressure was also found in the Fe-deficient FGT sample [77], similar to the FGT system. Overall, pressure modifies the metallic form of Fe_3−*x*_GeTe_2_ to its nonmetallic form.

### 4.4. Light Control

**Figure 11:**
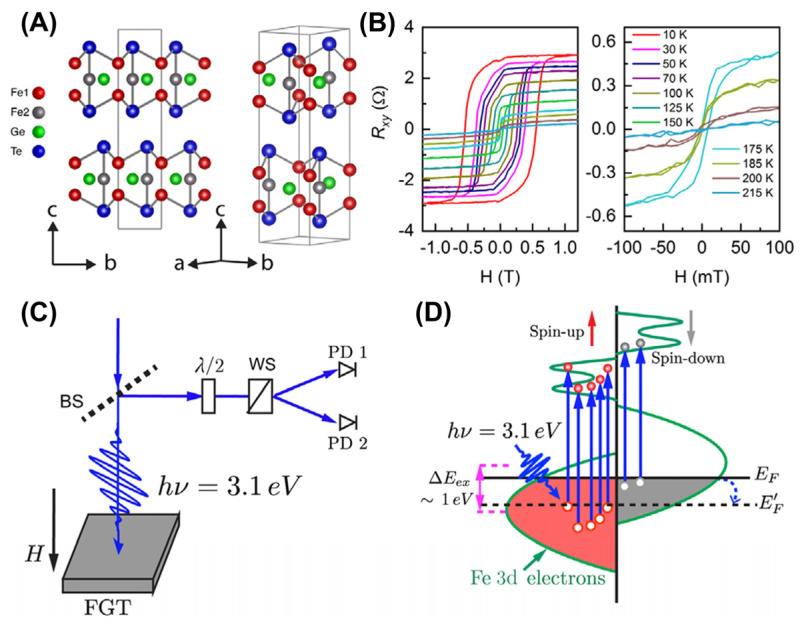
(**A**) The atomic structure of FGT monolayer. (**B**) Temperature–dependent Hall resistance (*R_xy_*) as a function of the perpendicular magnetic field. (**C**) Diagram of the experimental configuration for the polar magneto–optical Kerr effect (MOKE) measurements. BS and WS here represent the beam splitter and Wollaston splitter, respectively. (**D**) Schematic of the laser–excited DOS in few–layered FGT thin films. The photon energy of 3.1 eV causes electron transitions (vertical blue arrows) from occupied states below the Fermi level *E_F_* to the unoccupied states above *E_F_*. Reprinted with permission from [53]. Copyright 2020, American Physical Society.

The continuous modulation of monolayer transition-metal dichalcogenides (TMDs) without intrinsic magnetism, including MoS_2_ [157], WS_2_ [157], and WSe_2_ [158], has been achieved using the optical approach. Recently, Tengdin et al. [82] demonstrated that spin polarization was transferred from Mn sublattices to Co on the Heusler compound Co_2_MnGe via femtosecond laser pulse, which is closely related to the wave function of electrons before and after being excited by light. The ultrafast spin transfer caused by the instantaneous incident light on the material does not only occur in Co_2_MnGe, but is also a common feature of many materials. Notably, Xu et al. [53] reported that the magnetic anisotropy energy (MAE) and *T_C_* were mediated with a femtosecond laser pulse, as shown in Figure 11. The optical doping effect alters the electronic structure of FGT, thereby affecting exchange interactions, *T_C_*, and MAE. According to Figure 11B, the *T_C_* of FGT was estimated to be ~200 K. Under the excitation of a femtosecond laser, electrons transitioned from an occupied state to an unoccupied state, causing the Fermi level *E_F_* to shift downwards and crossing the enhanced density of states (DOS) shown in Figure 11D. Furthermore, some clear magnetic hysteresis loops at room temperature (RT) can be observed in FGT samples with different thicknesses, according to Polar-MOKE measurements (Figure 11C). The *T_C_* of FGT can be increased to above RT through light control, providing many opportunities for the development of spintronic applications for 2D magnets.

### 4.5. Electrical Control

**Figure 12:**
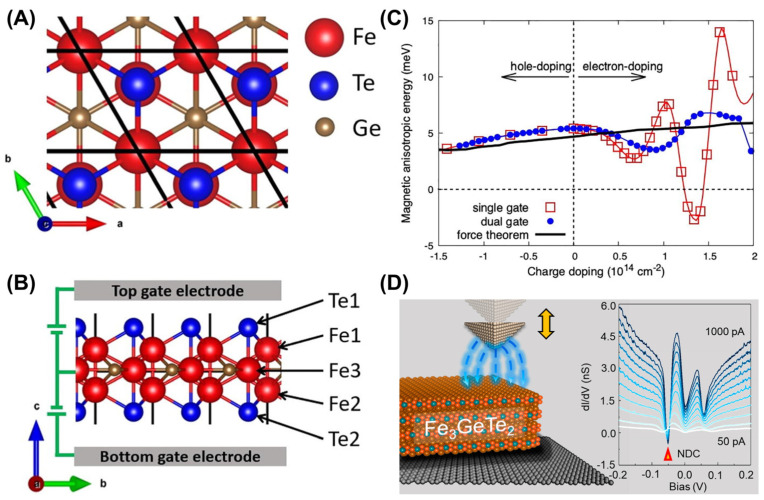
Schematics of the atomic structure (**A**,**B**) of the FGT monolayer. The dual–gate configuration for simulating the electrostatic gating is shown in (**B**). (**C**) The MAE per unit cell as a function of the charge doping concentration in the single–gate (squares) and dual–gate (dots) configurations. The results obtained using the force theorem are denoted by the black line. Reprinted from Ref. [83] with the permission of AIP Publishing. (**D**) A series of *dI*/*dV* curves taken from a set point of 50−1000 pA at the same position. Reprinted with permission from [84]. Copyright 2021 American Chemical Society.

Previous studies have shown that electric fields modify the magnetism of metal films [159,160,161] and Fe/MgO junctions [162] by influencing the behavior of the electrons. Recently, Wang et al. [83] calculated the effect of the electric field on the magnetic anisotropy of the FGT monolayer, as shown in Figure 12A–C. The effect of orbital splitting caused by electron doping on magnetic anisotropy was more pronounced; meanwhile, the influence of hole doping related to orbital occupation was relatively weak. In addition, the change in magnetic anisotropy (Figure 12C) was more obvious in the single-gate configuration (Figure 12B).

Additionally, the generation of negative differential conductance (NDC) [84] can also be driven by a local electric field in FGT (Figure 12D). Furthermore, the three peaks in the Fe *d* orbits underwent significant shifts under the electric field. As the electric field was enhanced, the off-plane FM of FGT weakened, resulting in a decrease in MAE. Remarkably, in single-layer FGT, the electric field induces charge transfer in the FGT monolayer in the field direction. Therefore, applying an electric field has become an effective way to mediate 2D FM.

### 4.6. Proximity Effects

Proximity effects [85,163,164,165,166,167] are another dominant area of the research into 2D materials. For example, by using 2D magnetic materials adjacent to a bulk semiconductor substrate [168] or 2D materials with strong spin–orbit coupling [169], their magnetism can be enhanced. Intriguingly, Zhang et al. [85] fabricated antiferromagnetic FePS_3_(FPS)/ferromagnetic Fe_3_GeTe_2_(FGT) heterostructures and detected the enhancement of *T_C_* and *H_C_* through proximity coupling effects. Furthermore, FPS/FGT/FPS exhibits a slightly different modulation of *H_C_* compared to FPS/FGT, which is related to AFM-FM coupling. Moreover, the long-range magnetic order induced by topology triggered by femtosecond laser pulses [57] could also be maintained at room temperature.

**Figure 13:**
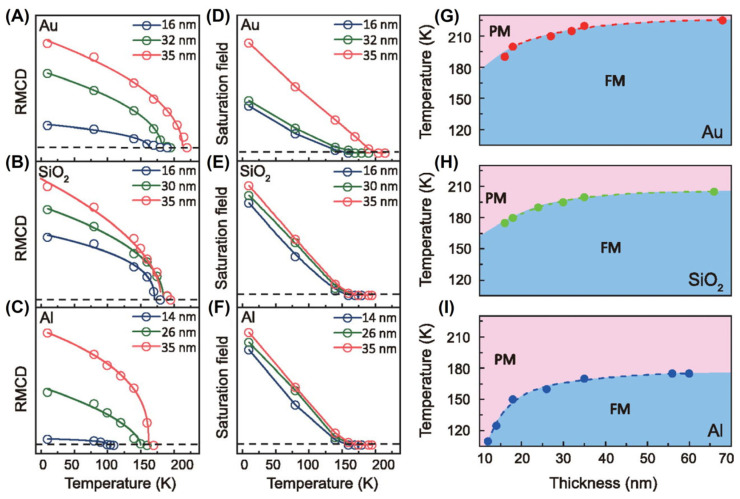
The RMCD signals of FGT flakes with varying thicknesses on Au, SiO_2_, and Al substrates are shown in (**A**–**C**), respectively. The saturation fields as a function of temperature for FGT flakes with varying thicknesses on three substrates are shown in (**D**–**F**). The empty circles are fitted using the function *α*(1 − *T*/*T_C_*)*^β^*. The dotted line corresponds to zero RMCD signal and saturation field. (**G**–**I**) *T_C_* was extracted as a function of the thickness of the FGT flakes on three substrates from RMCD measurements. Reprinted from [88], with the permission of AIP Publishing.

The aforementioned methods for mediating magnetism require additional equipment, such as a three-point-bending experimental setup [48,72], DAC, [78], femtosecond laser excitations [53], and the scanning tunneling microscopy (STM) tip [84]. Directly exfoliating FGT nanosheets [88] onto different substrates could also tailor magnetism, as shown in Figure 13. Intriguingly, FGT samples with different thicknesses all exhibited magnetism, while samples on different substrates exhibited different T_C_, indicating that the substrate has a modulation effect on T_C_ values. Furthermore, the lattice distortion and charge redistribution at the interface were related to substrate-induced FM, and this mechanism requires further exploration. Actually, substrates did not only affect the growth of 2D materials, but also determined their performance [170,171].

### 4.7. Doping Engineering

#### 4.7.1. Doping with 3*d* Transition-Metals

**Figure 14:**
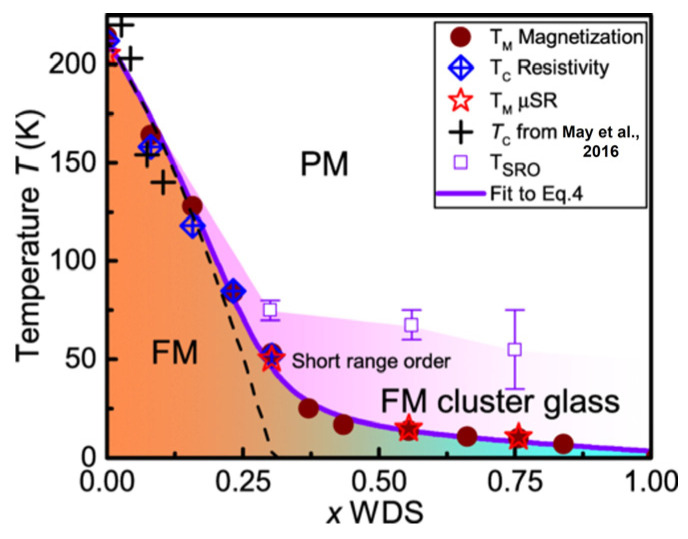
Phase diagram of a Ni-doped FGT single crystal determined by magnetization and µSR (*T_M_*), and resistivity measurements (*T_C_*) showing an FM region up to *x* = 0.3, which is smeared into an FM spin glass [9]. Muon spin relaxation and rotation measurements (µSR); Short-range order (SRO). Reprinted with permission from [38]. Copyright 2018, American Physical Society.

Doping 3*d*-transition metal atoms is an effective strategy for controlling magnetism [66,69,90,172,173,174,175,176]. Theoretical calculations have shown that almost all 3*d*-transition metal atoms (except for Co atoms) [97] are more inclined to replace Fe1 atoms (Figure 2A). The charge transfer generated by doping atoms weakens the magnetic moment of Fe atoms, while the weakening effect of Fe1 atomic magnetic moment is more significant. However, the magnetism increases after doping with Co atoms, which may be related to the shrinking of the a-axis lattice constant. In experiments, doping 3*d*-transition metal atoms in bulk single-crystal samples were usually achieved via CVT [93] or self-flux [38]. Doping Ni atoms suppressed the ferromagnetic order, which rapidly decreased with the increase in the doping amount. The *T_C_* decreased from 212 K to 50 K, and after reaching 0.44, the magnetic moment remained almost constant. Furthermore, the long-range magnetic order was suppressed and subsequently transformed into a glassy magnetic phase (Figure 14). However, doping Co atoms may cause an increase in *H_C_* and the appearance of hard magnetic phases; this is related to the movement of pinned domain walls [8].

**Figure 15:**
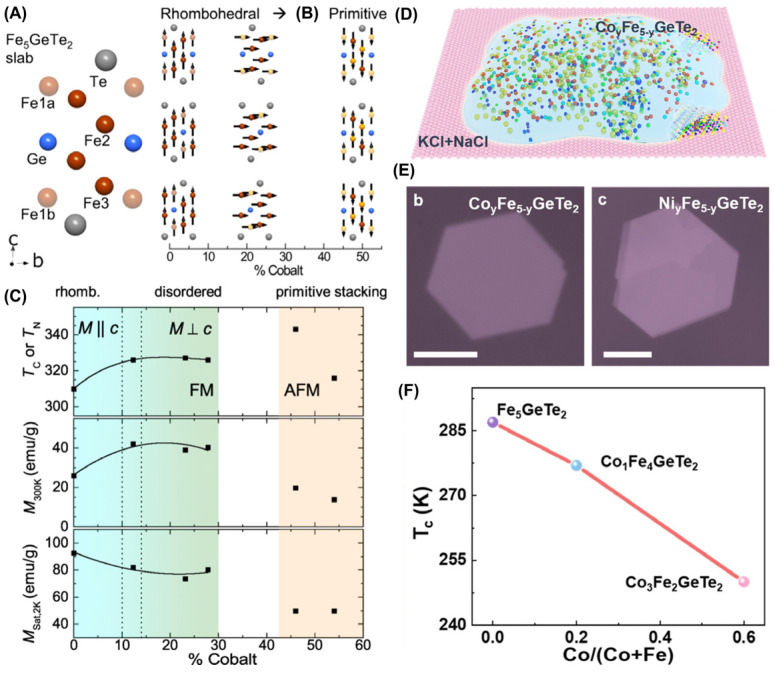
(**A**) Schematic of the F5GT layer with atomic types and positions labeled. (**B**) Change in the lattice type (rhombohedral/primitive) with cobalt doping (gold spheres) and the corresponding evolution of the magnetic order and anisotropy (black arrows). (**C**) Curie and Néel temperatures, magnetization induced along [001] at 300 K, and saturation magnetization. The dashed vertical lines indicate the approximate region where the magnetic anisotropy inverts for the ferromagnetic compositions; the solid lines in the FM region are intended to facilitate viewing. The AFM region is likely characterized by FM planes that are coupled antiferromagnetically along [001]. Reprinted with permission from [95]. Copyright 2020, American Physical Society. (**D**) Schematic of the melt flux containing Co*_y_*Fe_5−*y*_GeTe_2_. OM images of Co*_y_*Fe_5−*y*_GeTe_2_ (**E**) and Ni*_y_*Fe_5−*y*_GeTe_2_ (**E**). (**F**) Comparison of *T_C_* values of F5GT, Co_1_Fe_4_GeTe_2_, and Co_3_Fe_2_GeTe_2_. Reprinted with permission from [44]. Copyright 2022, American Chemical Society.

Bulk F5GT single crystals were also doped with Co atoms via CVT [95,96], as shown in Figure 15. As the amount of Co used for doping increases, it can drive the evolution of the lattice and of magnetism (Figure 15B). However, the nominal doping concentration was slightly different from the measured one, with only a specific concentration being more consistent. Afterward, Co atoms were doped into the lattice, resulting in a slight increase in their interlayer spacing. Indeed, a phase transition from FM to AFM occurred at high doses of doping in Figure 15C. However, Tian et al. [96] found that doping with 20% Co could increase its *T_C_* to 337 K and induce complex magnetic phase transitions at higher Co doping levels. Furthermore, hexagonal 2D Co*_y_*Fe_5−*y*_GeTe_2_ (Figure 15E) and Ni*_y_*Fe_5−*y*_GeTe_2_ nanoflakes (Figure 15E) were prepared via flux-assisted growth (Figure 15D) [44]. Various elements in the nanosheets were evenly distributed via energy dispersive spectroscopy (EDS) elements mapping, and there were significant differences in the energy spectra of samples doped with different amounts of Co, with a doping amount of up to 66.7%. Furthermore, the doping of Co atoms caused a decrease in *T_C_*, and as the doping amount increased, its *T_C_* decreased even further (Figure 15F). In addition, the magnetic anisotropy underwent significant changes. Similarly, Ni doping can also cause a decrease in *T_C_*. In other words, the higher the content of Fe, the higher its *T_C_*.

#### 4.7.2. Doping with Non-Metallic Atoms

Not only can Fe atoms be substituted with Co or Ni atoms [38,44,93,95,96,97], but doping can also be achieved by replacing Ge atoms with As atoms [92,98]. The doping of As atoms caused a decrease in the a-axis lattice constant and an increase in the *c*-axis lattice constant, thereby reducing the density of spin states below the Fermi level, resulting in a decrease in *T_C_* [92]. Furthermore, its *M_S_* decreased linearly with the increase in the doping amount in polycrystalline Fe_3−*y*_Ge_1−*x*_As*_x_*Te_2_ (0 ≤ *x* ≤ 0.85). Similarly, the expansion of the F5GT unit cell [98] in the c-axis direction and the contraction in the *ab* plane was also observed after doping with the As atom. In addition, its *T_C_* and *M_S_* decreased in polycrystalline Fe_5_Ge_1−*y*_As*_y_*Te_2_ (0 ≤ *y* ≤ 1), a phenomenon similar to that observed in the Fe_3-*y*_Ge_1−*x*_As*_x_*Te_2_ (0 ≤ *x* ≤ 0.85) samples. Moreover, the stacking disorder caused by the local AFM coupling can reduce its *M_S_*.

#### 4.7.3. Electron Doping

**Figure 16:**
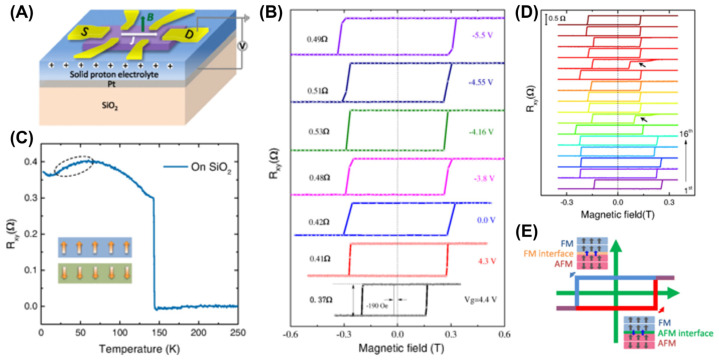
(**A**) Schematic of the Hall bar device on a solid proton conductor used for measurements in (**B**,**C**), in which the current density is *J* and a perpendicular magnetic field *B* is applied. (**B**) Gate–tuned FM in FGT nanoflakes. (**C**) Remanence Hall resistance *R_xy_* as a function of temperature. Inset: Schematic of a possible AFM phase in pristine FGT. (**D**) Effect of the exchange bias after zero–field cooling at 2 K. (**E**) Schematic of differing AFM–FM interfaces. Reprinted with permission from [100], Copyright 2020, American Physical Society.

Remarkably, Deng et al. [14] found that FGT devices could be operating in ionic gates, which provides a new approach for mediating 2D FM. Although they did not fully explain the relationship between it’s ferromagnetism and electron doping, the importance of this strategy was acknowledged. Soon after, gate-control was implemented to regulate magnetic resistance [99], magnetic phases [62,101], and interlayer coupling [100,102,177]. Furthermore, the *T_C_* and *H_C_* in FGT flakes [101] were decreased after Li^+^ doping from lithium-ion-conducting glass-ceramics (LICGC). In addition, electron doping influenced the Fe–Ge plane in the middle of the FGT monolayer, weakening it’s resistance and enhancing it’s *T_C_* [105]. As a typical example, the modulation of interlayer coupling was achieved by fabricating FGT hall devices on solid-state proton conductors (Figure 16A). Furthermore, they discovered a clear exchange bias (EB) when changing the gate voltage (Figure 16B), which may be related to the presence of an AFM phase at low temperatures (Figure 16C). However, a random exchange bias (Figure 16D) occurs after applying a higher gate voltage. Furthermore, the exchange bias and coercivity undergo a complex evolution with the measurement times, but there have also been cases of small EB values and large coercivity. In addition, the type of AFM–FM interface coupling determines the positive and negative exchange bias (Figure 16E).

**Figure 17:**
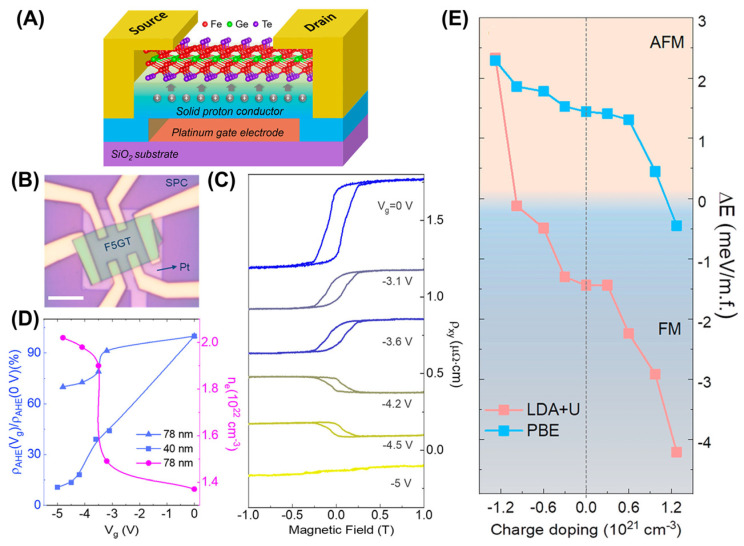
Schematic diagram (**A**) and optical image (**B**) of an F5GT SP–FET, where an F5GT flake lies on the solid proton conductor (SPC). Scale bar: 10 μm. (**C**) *ρ_xy_* loops. (**D**) Gate–voltage–dependent carrier densities and anomalous Hall ratios. (**E**) Evolution of the energy difference between FM and AFM (Δ*E*) with charge doping under LDA + U and PBE functionals. Reprinted with permission from [62]. Copyright 2021, American Chemical Society.

Additionally, Tan et al. [62] achieved the high-electron-concentration doping of F5GT through a solid proton conductor (Figure 17A,B). When a positive bias voltage was applied, the transport properties were unchanged. When a negative bias voltage was applied, there was a significant change in the transport properties caused by proton intercalation, especially when it reached −5 V, and the anomalous Hall loop disappeared (Figure 17C), accompanied by the appearance of a magnetic phase transition (Figure 17D,E). Moreover, different calculation methods have revealed that electron doping achieves a reversal of Hall conductivity and phase transition (Figure 17E). Therefore, electron doping or protonic gating indeed represents an efficient method of controlling magnetic phase transitions. Furthermore, Tang et al. [102] found that magnetic anisotropy in F5GT was continuously mediated by electrolyte gating. Moreover, the screening effect of itinerant electrons drives magnetic anisotropy to switch from an off-plane easy axis to an in-plane easy axis.

#### 4.7.4. Hole Doping

**Figure 18:**
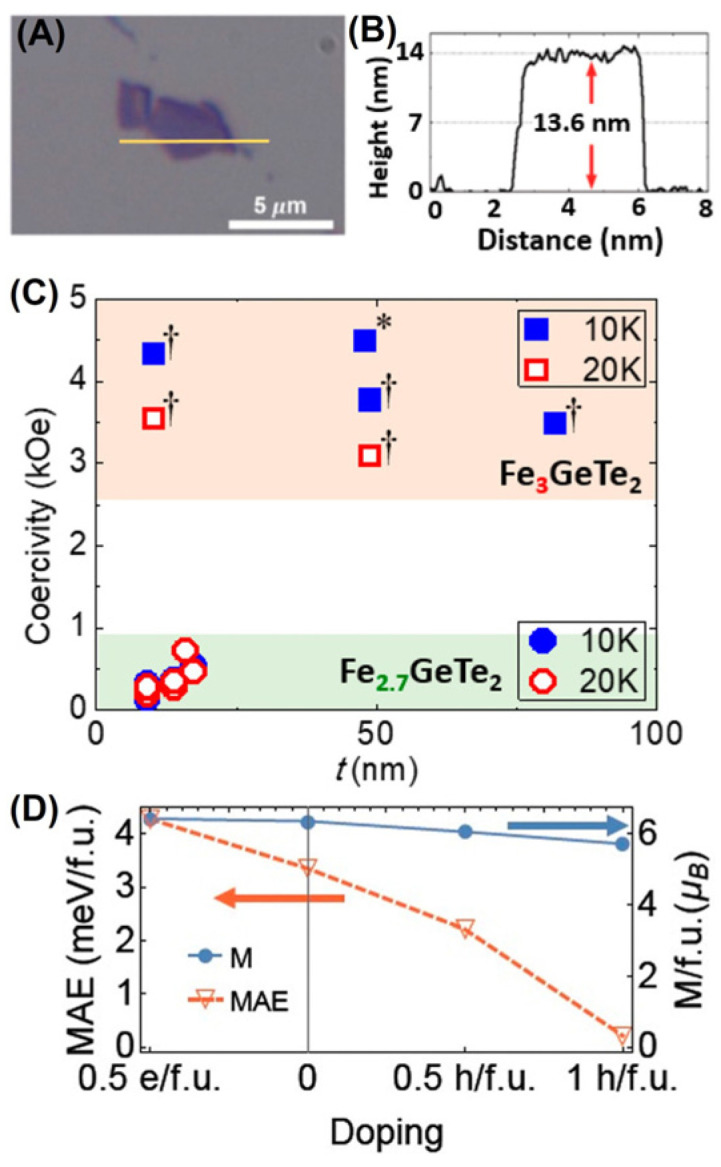
(**A**) Optical image of an FGT flake. (**B**) Height profile. (**C**) *H_C_* of Fe deficient Fe_3−x_GeTe_2_ and FGT. The Fe_3_GeTe_2_ data are taken from [15] [marked *] and [17] [marked †]. (**D**) MAE and doping-dependent magnetization. Reprinted with permission from [43]. Copyright 2019, American Chemical Society.

Inspired by the gate-mediated RTFM in FGT thin flakes [14], many attempts have been made to control its ferromagnetism through hole [43,94,106] or electron [43,94,105] doping. In particular, the magnetic anisotropy in exfoliated Fe_2.75_GeTe_2_ flakes (Figure 18A,B) was inhibited by hole doping, resulting in a decrease in *H_C_* (Figure 18C). The magnetic anisotropy could undergo a 93% attenuation, but the change in the magnetic moment was very small, as shown in Figure 18D. Furthermore, the electronic structure of Fe_2.75_GeTe_2_ single crystals changes due to hole doping, causing significant changes in magnetic anisotropy. In addition, another report [94] suggested that hole doping was beneficial for maintaining the long-range ferromagnetic order.

**Figure 19:**
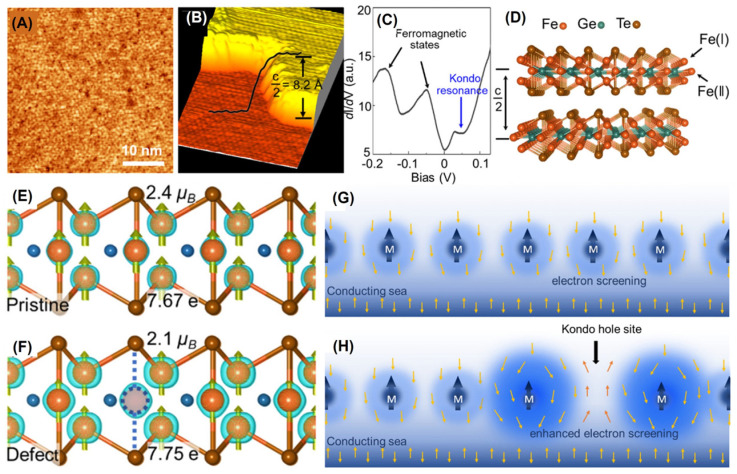
(**A**) Large–scale STM image of FGT. (**B**) Atom–resolved STM topography. (**C**) *dI*/*dV* curves. (**D**) Crystal structure of FGT. (**E**,**F**) Side views of the crystal structures for these two situations. (**G**,**H**) Illustrate the Kondo screening in a pristine Kondo lattice and in a lattice with a Kondo hole, respectively. Reprinted with permission from [106]. Copyright 2021, American Chemical Society.

Remarkably, the intrinsic Fe vacancies [106] were probed via STM, as presented in Figure 19A–D. The peak near 20 mV originated from the Kondo lattice [106,121], and this Fe vacancy is called a Kondo hole (Figure 19C,D). Hole doping elevated the energy band, and the Fermi surface of FGT was shifted towards a lower energy level. After the formation of Fe vacancies at the Fe (II) site, the magnetic moment near the Fe (I) site decreased (Figure 19E,F), accompanied by the appearance of a higher charge density. Furthermore, the introduction of Fe vacancies reduced the magnetic moment near them, further strengthened the Kondo screening effect, and, thus, weakened the magnetism, as detailed in Figure 19G,H. The Kondo holes can affect the charge distribution of their own sites, and converting them into momentum space has a more significant impact. In other words, hole doping weakened the ferromagnetism of FGT.

### 4.8. Intercalation or Irradiation

**Figure 20:**
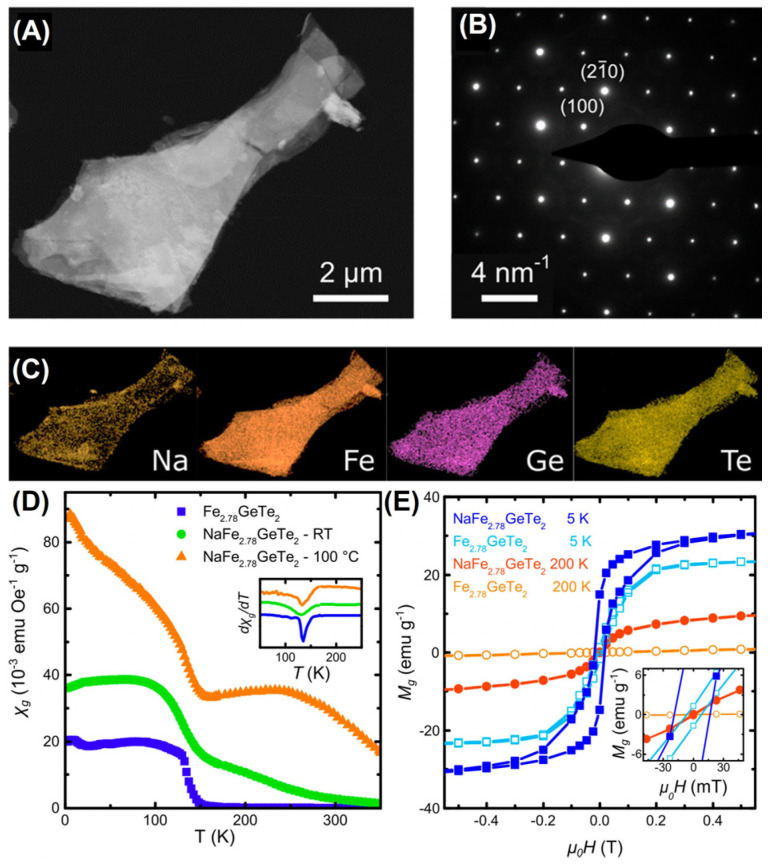
(**A**–**C**) TEM image and representative SAED pattern of NaFe_2.78_GeTe_2_ as well as EDX maps of the constituting elements. (**D**) Temperature dependence of the specific magnetic susceptibility *χ_g_* at *μ*_0_*H* = 0.01T. (**E**) *M*–*H* curves. Reprinted with permission from [107]. Copyright 2019, American Chemical Society.

Recently, inserting sodium into Fe_2.78_GeTe_2_ powders [107] can raise its T_C_ to ~300 K, as shown in Figure 20. After intercalating Na, more exposed edges appeared, and their layered features remained unchanged in a single crystal structure (Figure 20A,B). More specifically, the Fe, Ge, and Te elements (Figure 20C) were evenly distributed in the sample, while the inserted Na was concentrated at the edge. A phase transition occurred from PM (Fe_2.78_GeTe_2_) to FM (NaFe_2.78_GeTe_2_) at 200 K (Figure 20D), and the M_S_ was enhanced (Figure 20E). Furthermore, the magnetic hysteresis loops are also measured at 350 K. Notably, impurity phases, such as Fe or Fe_2−x_Ge, dominated the RTFM in the NaFe_2.78_GeTe_2_ samples. Alternatively, the T_C_ and exchange bias could be mediated with Fe-intercalation [108], which induces magnetic order by reinforcing magnetic coupling. However, the detected Te_Ge_ antisite defects had no modulation effect on the T_C_ of different samples. Thus, Na intercalation provides a novel strategy for enhancing T_C_, which is related to the tensile strain.

Inspired by pattern-induced ferromagnetism [16], Yang et al. [109] also improved the *T_C_* of FGT to 450 K via Ga irradiation. Irradiation induces an amorphous structure on the surface of the sample, which facilitates the formation of magnetic vortex states in FGT micropatterns. Moreover, irradiation causes a magnetization transition.

### 4.9. Twisting

Twisting 2D materials can introduce some novel properties, such as magnetism [178,179] and superconductivity [180], which trigger the interaction topology with magnetism in 2D ferromagnets, resulting in the formation of skyrmions [181,182] or magnons [183,184] in the twisting system. In fact, the stacking order directly affects the magnetism of bilayer CrI_3_ by changing the crystal structure [178] or interlayer magnetic coupling [178]. Surprisingly, the magnetism was obtained in double bilayer CrI_3_ [185] at small twist angles. Although the phase transition from AFM to FM has been theoretically achieved in twist-stacking bilayer FGT [110,179], it has not yet been experimentally achieved [186].

### 4.10. Patterning

**Figure 21:**
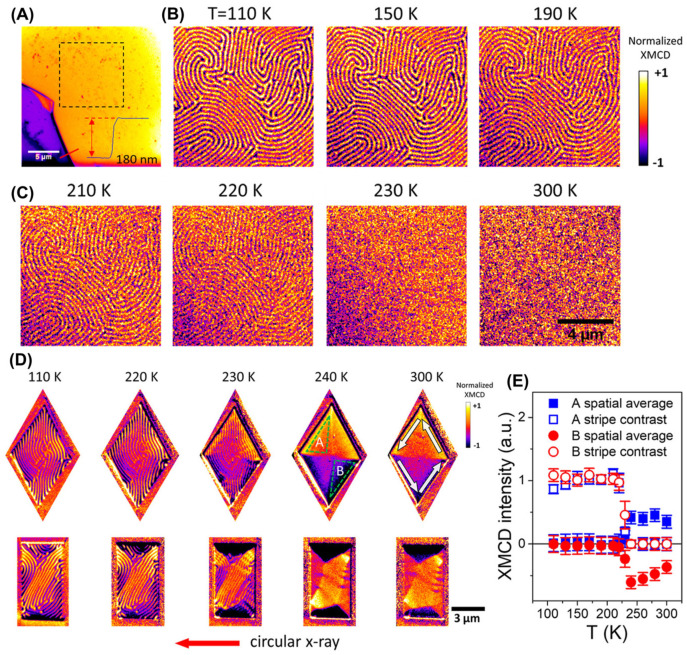
(**A**) PEEM topography image of an FGT flake (golden color) on a silicon substrate (purple color). (**B**,**C**) Magnetic–stripe domains. (**D**) Micron–sized diamond–shaped and rectangular–patterned structures. The magnetic stripe contrast (out-of-plane magnetization component, labeled as A and the spatially averaged contrast (in-plane magnetization component, labeled as B from the two selected areas. (**E**) Temperature dependence of the magnetic stripe contrast (out–of–plane magnetization component) and the spatially averaged contrast (in–plane magnetization component) from the two selected areas. Reprinted with permission from [16]. Copyright 2019, American Chemical Society.

Magnetic domain patterns on FGT surfaces can be modulated with various mechanisms [8,13,187,188], one of which is the phase transition from FM to AFM related to interlayer coupling [13]. The photoemission electron microscopy (PEEM) image in Figure 21A–E clearly shows the magnetic domain structure of FGT nanosheets, and the stripe domain structure disappears after reaching the T_C_ of 230 K. After patterning the FGT sample into diamond and rectangular shapes using a focused ion beam (FIB), striped magnetic domain structures, similar to those in the unpatterned FGT (Figure 21B,C), were also observed, as shown in Figure 21D. However, the striped domain structure did not completely disappear and was significantly weakened at 230 K (Figure 21C).

In addition, there were only in-plane magnetic domains in the patterned FGT at 240 K (Figure 19D); they disappeared at ~370 K, indicating that the *T_C_* of bulk FGT was increased to 370 K. Notably, a novel magnetic vortex state or magnetic multidomain state was developed in patterned FGT (Figure 19D) at 300 K. Furthermore, spin reorientation occurred with increasing temperatures (Figure 19E). When using FIB to construct FGT patterns, Ga ions were unintentionally implanted into the sample, which may have caused the increase in *T_C_*; however, to date, this hypothesis remains speculative.

## 5. Band Structure of Ferromagnetic Fe*_x_*GeTe_2_

**Figure 22:**
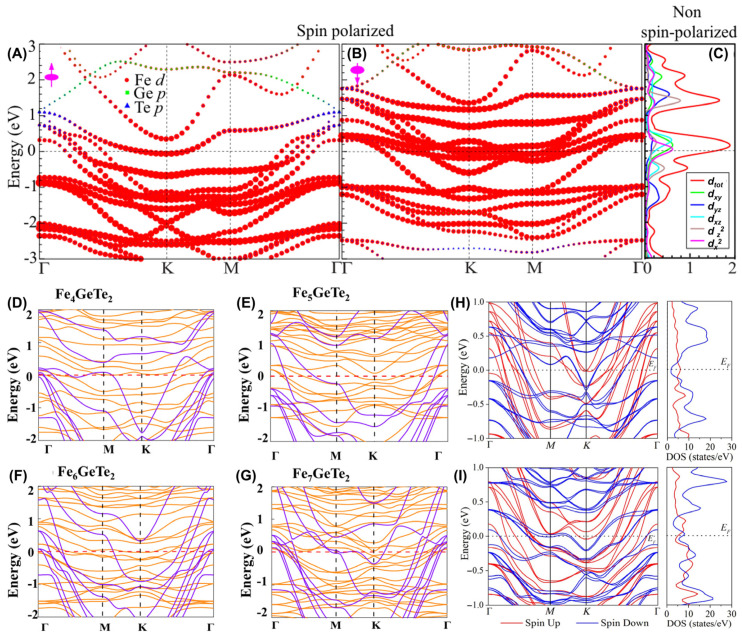
(**A**,**B**) DFT–LDA–calculated band structures of the FGT monolayer. Electronic density of states (DOS) for the Fe d states in the non–spin–polarized system in units of states/eV/Fe atom/spin of FGT monolayer. This non–spin–polarized electronic structure (**C**) is used to obtain the density of states at the Fermi level D(*E_F_*). Reprinted with permission from [46]. Copyright 2016, American Physical Society. (**D**–**G**) DFT + U–calculated band structures of Fe*_x_*GeTe_2_ (4 ≤ *x* ≤ 7). The purple and orange lines are spin-up and spin-down bands, respectively. Reprinted with permission from [64]. Copyright 2022, Springer Nature. Spin–resolved band structures and DOS of F4GT (H) bilayer (BL) (**H**) and F5GT (**I**) BL. The Fermi level is set to zero. Reprinted with permission from [190]. Copyright 2023, American Physical Society.

Like its bulk form, the FGT monolayer is metallic, as shown in Figure 22A–C. Its band structures near the Fermi level can mainly be attributed to the contribution of the Fe *3d* orbitals. Moreover, it was confirmed that the FGT monolayer has the itinerant FM order according to Stoner’s criterion [46,189]. Remarkably, the Stoner model related to itinerant electrons can be used to better elucidate the spontaneous magnetization in most 2D metallic ferromagnets. In addition, the electronic band structures of all the Fe*_x_*GeTe_2_ systems are metallic (Figure 22D–G), similar to the FGT monolayer.

Furthermore, the F4GT and F5GT bilayer [190] have band structures similar to the FGT bulk and FGT monolayer, exhibiting metallic magnetic properties. However, there is a significant difference in the polarizability of F4GT and F5GT near the Fermi level, which leads to their different transport characteristics. The unique nature of FGT gives its related devices many advantages, including nonvolatility, low reversal magnetic field, and the magnetic field reversal Fe*_x_*GeTe_2_ electrodes through the spin-polarized current.

## 6. Fe*_x_*GeTe_2_-based Devices

**Figure 23:**
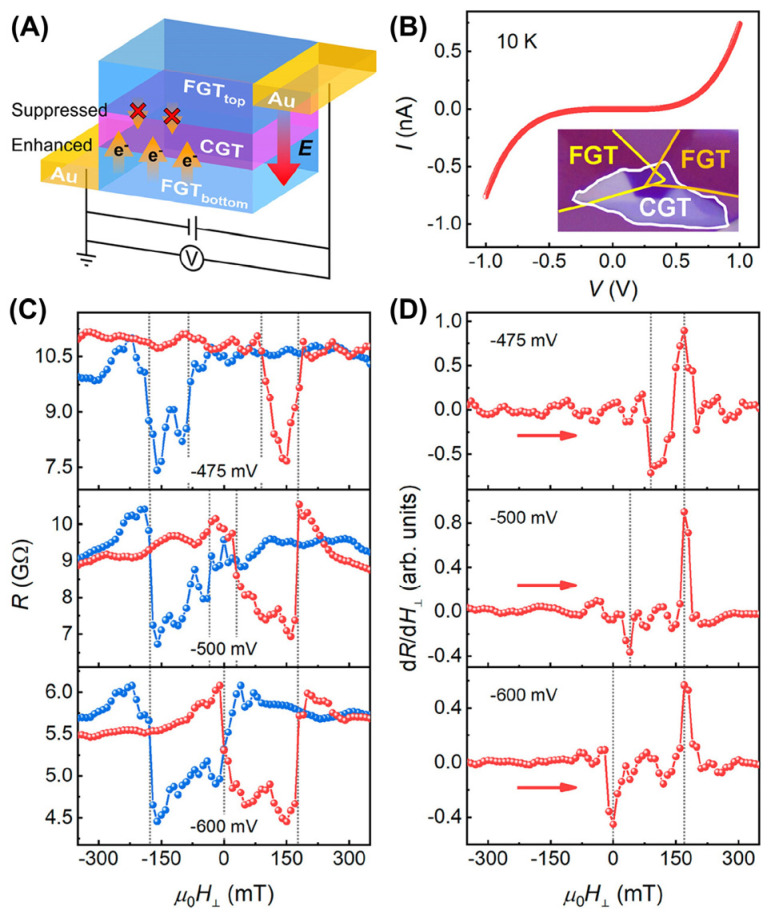
(**A**) Schematic of an FGT/CGT/FGT MTJ and the effect of directional electric fields on the charge transfer. (**B**) *I*–*V* curve. The inset is the optical image of this FGT/CGT/FGT MTJ. (**C**) Out–of–plane magnetic–field–dependent resistance, after subtracting the noise background. The gray–dotted lines indicate the positions of the switching fields. The red (blue) curves present the magnetic field sweeping from negative (positive) to positive (negative). (**D**) Numerical derivative (*dR*/*dH_⊥_*) curves under negative *V* bias. Reprinted with permission from [115]. Copyright 2023, American Chemical Society.

To the best of our knowledge, four typical devices have been constructed based on metallic Fe*_x_*GeTe_2_ ferromagnets, including magnetic tunnel junctions (MTJ) [112,113,114,115,136], tunneling spin valves [18,99,116,117], nonlocal spin valves [118] and spin–orbit torque (SOT) devices [20,119,191]. As shown in Figure 23A,B, a nonlinear behavior originating from tunneling characteristics [115] was exhibited in the *I*–*V* curve. Furthermore, a typical spin-valve behavior was also identified in the hysteresis loops (Figure 23C,D). After applying a specific voltage, the spin-transfer torque (STT) generated by the current caused the bottom FGT electrode to switch, which was closely related to MAE.

**Figure 24:**
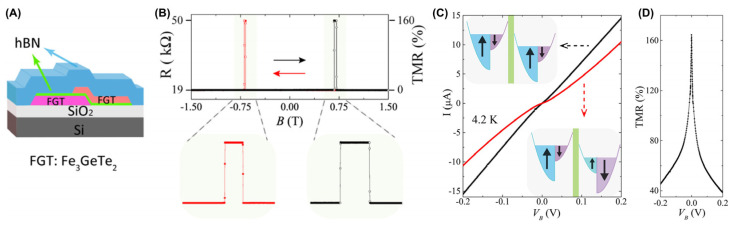
(**A**) Spin–valve effect in an FGT/hBN/FGT van der Waals heterostructure. (**B**) Tunneling resistance measured at T = 4.2 K with B applied parallel to the FGT c–axis. Very sharp–resistance jumps are observed for B ≈ ± 0.7 T. The variation in tunneling magnetoresistance (TMR) is ∼160%. Upper panels: zoomed–in view of the magnetoresistance. (**C**) *I*–*V* curves measured with the magnetization in the two FGT electrodes pointing parallel (black curve, B = 0 T) and antiparallel (red curve, B = −0.68 T) to each other. The insets show the corresponding configuration in the density of states of majority and minority spins in the two electrodes. (**D**) Bias dependence of TMR. Reprinted with permission from [18]. Copyright 2018, American Chemical Society.

**Figure 25:**
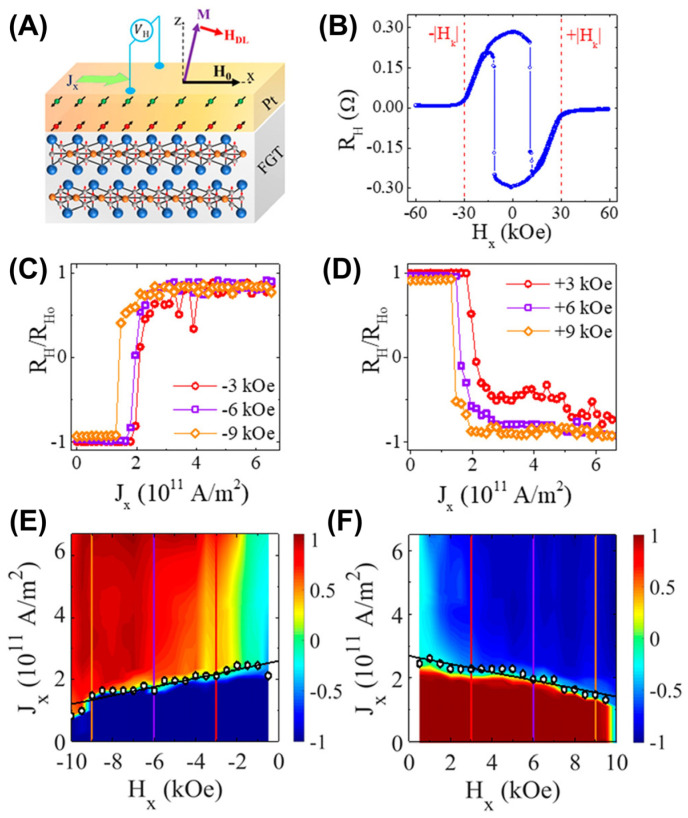
(**A**) Schematic illustration of the effective field responsible for switching the magnetic state of FGT in FGT/Pt hybrid devices. *J_x_* is the injected current density, *H_x_* is the applied in-plane field, *H_DL_* is the effective field from damping–like spin–orbit torques (SOT), and *M* is the FGT’s magnetization. (**B**) Hall resistance for our FGT(15 nm)/Pt(5 nm) device with anisotropy field *H*_k_ labeled on the graph. (**C**–**F**) Effective switching current as a function of the applied in–plane negative (**E**) and positive (**F**) bias field. The color scale represents the switching resistance as a percentage of the absolute value of the anomalous Hall resistance at zero current *R*_H0_. (**C**,**D**) correspond to the line cuts in (**E**,**F**). Reprinted with permission from Ref. [20]. Copyright 2019, American Chemical Society.

Regarding another typical device, Wang et al. [18] observed tunneling spin-valve behavior (Figure 24A,B) in an FGT/*h*BN/FGT heterostructure (Figure 25A). Its TMR reached up to 160%. The spin of the tunneling electrons created a nonlinear bias-dependent *I*–*V* curve related to Figure 24C. As the bias voltage increased, TMR exhibited a highly significant attenuation, as shown in Figure 24D. The inelastic tunneling channel related to bias led to spin relaxation, which may suppress TMR signals.

After applying a voltage to FGT/Pt hybrid devices (Figure 25A) [20], a current was generated between FGT and Pt, forming spin–orbit torques in Pt. Furthermore, a hard magnetic loop (Figure 25B) similar to an FGT device has also been observed in FGT/Pt devices. As the applied in-plane magnetic field *H_x_* was increased, its transition current decreased (Figure 25C–F), regardless of the direction of *H_x_*. This switch was related to the magnetic domain and domain walls. Moreover, the low switching current of the FGT monolayer was beneficial for exploring more effective devices. Very recently, Wang et al. [191] increased the *T_C_* of FGT to RT by the topological insulator Bi_2_Te_3_ and achieved the SOT-driven magnetization switching at RT.

More significantly, a nonlocal spin valve device [118] that can operate at RT has also been successfully constructed. In addition, highly efficient spin manipulations were observed at the F5GT/graphene interface.

## 7. Magnetic Skyrmions in Metallic Fe*_x_*GeTe_2_

As topological magnetic materials, the spin textures in Fe*_x_*GeTe_2_ are also regulated by the Fe stoichiometry. For instance, Bloch-type skyrmion bubbles [192] were observed in FGT by using Lorentz transmission electron microscopy (LTEM). However, in another study [193], Néel-type skyrmions were reported, which indicated the existence of Dzyaloshinskii-Moriya interaction (DMI) in FGT. Generally, FGT was considered as a centrosymmetric material in the space group *P*6_3_/*mmc* [35], which should not possess the asymmetric DMI. A possible interpretation is that the asymmetric interface between the pristine and oxidized FGT may induce an interfacial DMI [193].

More recently, a comprehensive study was implemented to answer this question more clearly [194]. It is found that the crystal structure of Fe_3−*x*_GeTe_2_ can be tuned by the Fe stoichiometry, that is, Fe_3−*x*_GeTe_2_ changes from centrosymmetric *P*6_3_/*mmc* space group to non-centrosymmetric *P*6_3_*mc* space group when x is larger than ~0.2 [194]. Notably, the *P*6_3_*mc* space group allows an in-plane DMI in Fe_2.74_GeTe_2_, leading to the formation of Néel-type skyrmions. Whereas in Fe_3.00_GeTe_2_, the centrosymmetric *P*6_3_/*mmc* structure forbids the DMI. Thus, Bloch-type skyrmion bubbles were observed when the dipole–dipole interaction and magnetic anisotropy obtained a delicate balance.

Moreover, in the *ab*-plane of F5GT, Bloch-type skyrmion bubbles with unconventional helicity polarization were observed [195,196], which shows special application potential for data storage and processing using the helicity freedom of skyrmions in the future. Additionally, in the *ac* and *bc*-plane of F5GT, (anti)meron chains were observed [197]. The tunable topological spin textures pave a promising way for constructing novel spintronic devices using Fe*_x_*GeTe_2_.

## 8. Outlook

In this review, we introduced the developments in and structures of 2D metallic Fe*_x_*GeTe_2_ ferromagnets. Then, we summarized six experimental methods, ten FM modulation strategies, and four typical spintronic devices that use 2D Fe*_x_*GeTe_2_ materials. Finally, we outlined the challenges and potential research directions in this field.

The samples prepared using the SSR, CVT, and flux methods were mainly bulk single crystals. Since the successful exfoliation of mono- or few-layer FGT, 2D Fe*_x_*GeTe_2_ has only been obtained experimentally. Very recently, CVD and MBE have also been used to prepare these materials. However, the lateral dimension of samples obtained via CVD was small and it was very difficult to accurately control the number of layers. Although MBE can be used to prepare wafer-scale materials and achieve 2D mode growth, it requires a high vacuum environment and is costly. So far, there is still no cost-effective method for preparing wafer-scale materials with controllable layers. In addition, new experimental methods, such as substitution reactions [198], are also worth further exploration.

Although ten strategies for regulating ferromagnetism have been proposed, not all of them have been achieved experimentally. Among these methods, twisting was only achieved through theoretical regulation. It is thus necessary to develop more strategies that could be executed experimentally to obtain RFTM in 2D Fe*_x_*GeTe_2_ materials. Furthermore, it would also be highly valuable to obtain FGT materials with good air stability [199] via new methods, as this would provide more convenient conditions for fabricating devices [200].

## Data Availability

Not applicable.

## Abbreviations

2D	Two-dimensional
3D	Three-dimensional
AFM	Antiferromagnetism
ATMs	Atomically thin materials
CGT	Cr_2_Ge_2_Te_6_
CS-CVD	Confined space chemical vapor deposition
CVD	Chemical vapor deposition
CVT	Chemical vapor transport
DFT	Density functional theory
DMI	Dzyaloshinskii–Moriya interaction
DOS	Density of states
EB	Exchange-bias
EDXS	Energy-dispersive X-ray spectroscopy
EDS	Energy dispersive spectroscopy
EDX	X-ray spectroscopy
FAG	Flux-assisted growth
FGT	Fe_3_GeTe_2_
F4GT	Fe_4_GeTe_2_
F5GT	Fe_5_GeTe_2_
FIB	Focused ion beam
FM	Ferromagnetism
FPS	FePS_3_
GGA	Generalized-gradient approximation
HAADF	High-angle annular dark-field
HRTEM	High resolution transmission electron microscopy
LDA	Local density approximation
LDA + U	Local density approximation plus Hubbard U
LICGC	Lithium-ion conducting glass-ceramics
LR	Long-range
LRFO	Long-range ferromagnetic order
LRMO	Long-range magnetic order
LTEM	Lorentz transmission electron microscopy
MAE	Magnetic anisotropy energy
MBE	Molecular beam epitaxy
MC	Monte Carlo
MCD	Magnetic circular dichroism
MFT	Mean field theory
MOKE	Magneto-optical Kerr effect
MTJ	Magnetic tunnel junctions
NDC	Negative differential conductance
PBE	Perdew–Burke–Ernzerhof
PEEM	Photoemission electron microscopy
PET	Polyethyleneterephthalate
RHEED	Reflection high-energy electron diffraction
PI	Polyimide
PM	Paramagnetism
PRA	Random phase approximation
PVA	polyvinyl alcohol
p-xrd	Powder X-ray diffraction
RT	Room temperature
RTFM	Room-temperature ferromagnetism
SAED	Selected area electron diffraction
Sc-xrd	Single-crystal X-ray
SEM	Scanning electron microscopy
SQUID	Superconducting quantum interference device magnetometry
SOC	Spin-orbit coupling
SSR	Solid-state reaction
STEM	Scanning transmission electron microscopy
STM	Scanning tunneling microscopy
STT	Spin-transfer torque
TMDs	Transition-metal dichalcogenides
TMR	Tunneling magnetoresistance
TS-LPE	Three-stage sonication-assisted liquid-phase exfoliation
XRD	X-ray diffraction
*R*	Bending radius
*T*	Film thickness
*T*_C_	Curie temperature
*J*	Exchange coupling constant
*J_x_*	the injected current density
Δ*E*	Total energy difference
*ε*	the applied strain
